# Role of MRI in the diagnosis and evaluation of cavernous hemangioma of the arm

**Published:** 2014-03-25

**Authors:** ME Ciurea, S Bondari, LE Stoica, IA Gheonea

**Affiliations:** * Department of Plastic and Reconstructive Surgery, Craiova University of Medicine and Pharmacy; ** Radiology and Imaging Department, Craiova University of Medicine and Pharmacy; *** Dermatology Department, Craiova University of Medicine and Pharmacy

**Keywords:** cavernous hemangioma, MRI, angioCT

## Abstract

Abstract

Sinusoidal hemangioma is a rare type of cavernous hemangioma with different clinico-pathological aspects. They are usually localized in the extremities with interest in the subcutaneous layer. The new imaging techniques play an important role in diagnosis, evaluation and follow-up of these types of tumors. We describe the case of a 21-year-old patient, four times operated for a recurrent soft tissue tumor, located intramuscularly in the distal third of the upper limb. Plain X-ray and computer tomography (CT) showed a nonspecific mass with calcification. The MRI (magnetic resonance imaging) exam demonstrated a lobulated heterogeneous signal tumor mass in the biceps brachial muscles, with high signal intensity on T2 weighted images and intermediate signal on T1 weighted images. MRI accurately assessed the extent of the tumor and evaluated the recurrence. MRI imagings combined with contrast-enhanced sequences were used to classify the lesions in low flow vascular disorders. CT angiography with multiplanar reconstructions (MPR), maximum intensity projections (MIP) and volume-rendered reconstructions (VR) was useful in confirming the venous origin of the tumor.

## Introduction

Hemangiomas are the most common benign tumors of soft tissues and the intramuscular types comprise 0.8% of all cases [**1**]. Hemangiomas are histopathologically divided in capillary, cavernous, arteriovenous, venous, and mixed variations [**2**]. Sinusoidal hemangioma represents a rare subtype of cavernous hemangiomas, which presents with different clinico-pathological aspects. Magnetic resonance imaging (MRI) plays an important role in the assessment of these lesions, concerning the diagnosis the type of lesions and evaluation of the extent, for proper treatment guidance. Also MRI has emerged as a noninvasive diagnostic tool in postsurgical and recurrence evaluation of hemangiomas [**3**].

## Case presentation

We present the case of a 21-year-old female patient who presented in our Radiology and Imaging Department with the appearance of two subcutaneous swellings in her right arm at the extremities of a scar from previous surgical interventions. The history and clinical data has shown that the patient was diagnosed in childhood with hemangioma of the arm, with the onset at 7 years old and six recurrences within 14 years. At 7 years old, she presented with a skin tumor located in the lower third of the right arm, which was surgically removed. The histopathology diagnosis was hemangioma. Three months after the surgery, the tumor recurred for the first time. After 9 years, the patient accused swelling and local pain and was subjected to another surgery for tumor excision, with a favorable postoperative evolution. After three months, another subcutaneous formation appeared, without changes in skin coverings, which grew in size and was slightly painful to effort. Another recurrence of the tumor for which the patient underwent surgical excision was at the age of 18 years and three months after. 

The last recurrence was at 19 years old, when the clinical examination revealed the presence of postoperative local dehiscence upon which there was a soft tissue tumor, measuring 10 cm in large diameter. The patient accused local pain exacerbated by exercise, the increase in tumor size during exercise and paresthesia. 

**Fig. 1:**
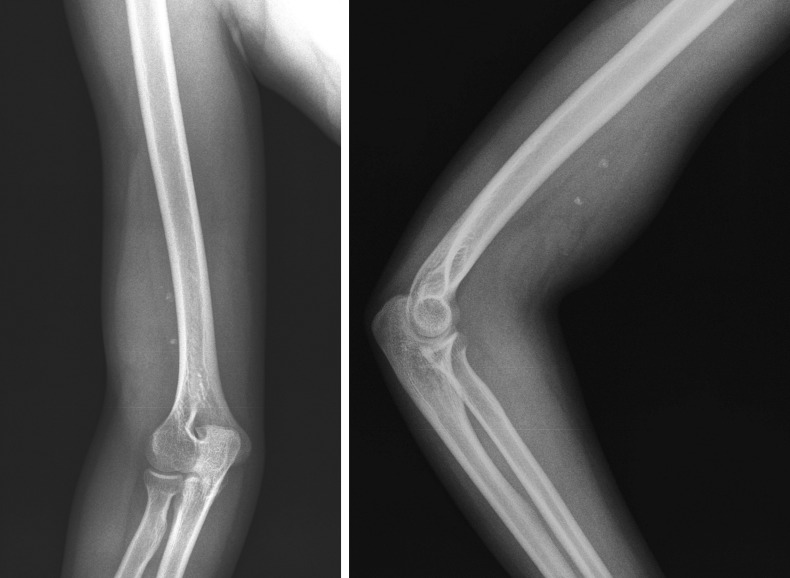
Plain upper limb X-ray in antero-posterior (a) and lateral (b) view showing a swelling of the lower third of soft tissues with calcification and intact bone structures

 The plain X-ray revealed a nonspecific soft-tissue mass in the lower third of the upper right limb with calcification inside, without any changes in the adjacent bone structures (**Fig. 1 a,b**). 

**Fig. 2:**
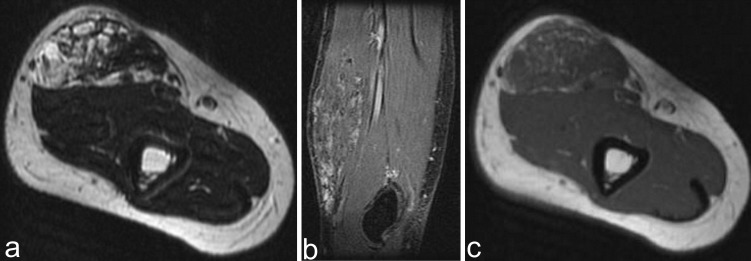
1.5T MRI showing lobulated and septated tumor in the biceps brachial muscles, with high signal intensity on T2 weighted and fat suppression images with calcification (a,b); intermediate signal on T1 weighted images (c)

The MRI exam demonstrated round-oval heterogeneous signal tumor mass in the biceps brachial muscles, with high signal intensity on T2 weighted images and septa (**Fig. 2a**). On T1 weighted images, the tumor had an intermediate signal with diffuse areas of fat increased signal (**Fig. 2b**). On T2 fat-sat sequences, punctuate signal voids areas related to phleboliths can be seen (**Fig. 2c**). The imaging features were specific for the recurrence of hemangioma, therefore gadolinium was not administered.

**Fig. 3:**
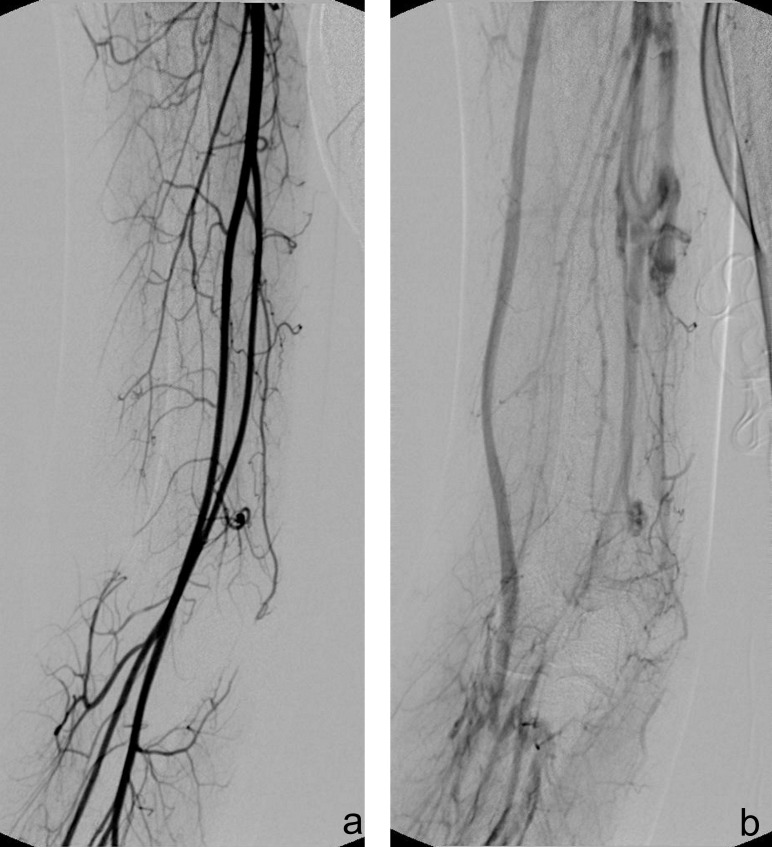
Angiography demonstrated the intact upper limb arterial supply in the arterial phase (a), and the dilatation of basilic and cephalic vein, with abnormal venous vessels (b)

A classic angiography was also performed, which demonstrated the lack of connection with the arterial structures, in particular with brachial artery, and the dilatation of basilic and cephalic vein. Also this technique revealed the abnormal venous vessels inside the tumor (**Fig. 3a,b**).

The therapeutic strategy included an enlarged tumor excision and exploration of brachial axis. The surgical exploration was very difficult due to the adhesion of tumor with brachial biceps and it sustained the hypothesis of venous vascular malformation. Histopathological examination of surgical sample included usual techniques for inclusion in paraffin and Hematoxylin–Eosin stained and also immunohistochemical analysis. The final diagnosis was sinusoidal hemangioma of the arm.

Currently, the patient presented with swelling in the upper and lower third of her arm, accusing pain in axilla and breast. The physical examination revealed two bulging in her arm, of elastic consistency, mobile on superficial anatomical plans, with a slightly irregular surface and without changes in the covering skin.

**Fig. 4:**
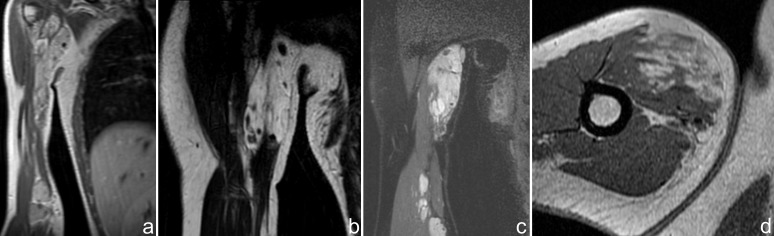
MRI exam showing two large soft tissue masses, localized in the upper and lower brachial muscles, with the same signal intensities on T1 and T2 sequences (a,b) and extension in the anteromedial axillary zone (b). Similar areas with high signal on STIR sequences were observed between these two tumors (c). The tumors enhance heterogeneous with signal voids corresponding to calcifications (d)

The MRI exam showed two large soft tissue masses, localized in the upper and lower brachial muscles, with intermediate signal intensity relative to muscle on T1 weighted images (**Fig. 4a**) and high-signal-intensity lesion on T2-weighted MR image (**Fig. 4b**). The upper lesion advanced in the anteromedial axillary zone. The tumors had septa and also punctate low signal-intensity areas, corresponding to calcification. Round-oval areas with similar MR features were observed between these two tumors (**Fig. 4c**). After contrast media administration, the tumors had heterogeneous enhancement with numerous signal voids corresponding to calcifications (**Fig. 4d**).

**Fig. 5:**
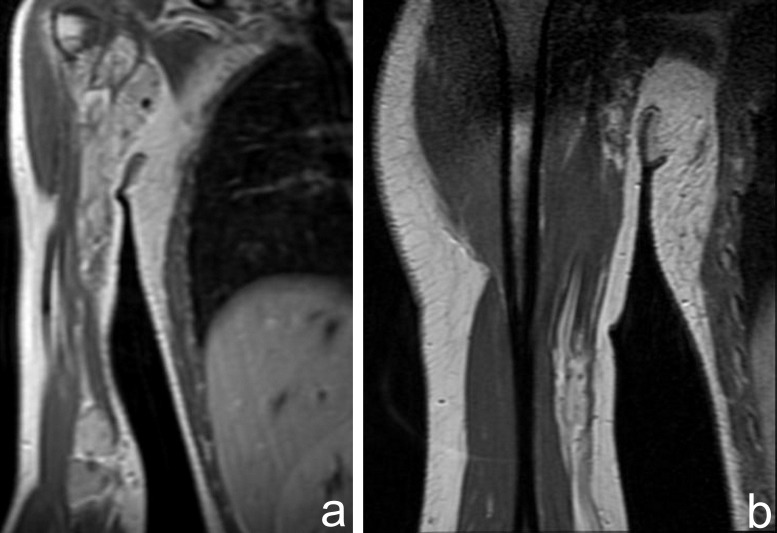
Late venous uptake of the lesions after contrast media administration (a) and the connection to an ectatic vein (b) are revealed on post contrast coronal T1 sequences

The post contrast sequences evidenced late venous enhancement of the lesions and the connection to an ectatic vein (**Fig. 5a,b**).

**Fig. 6:**
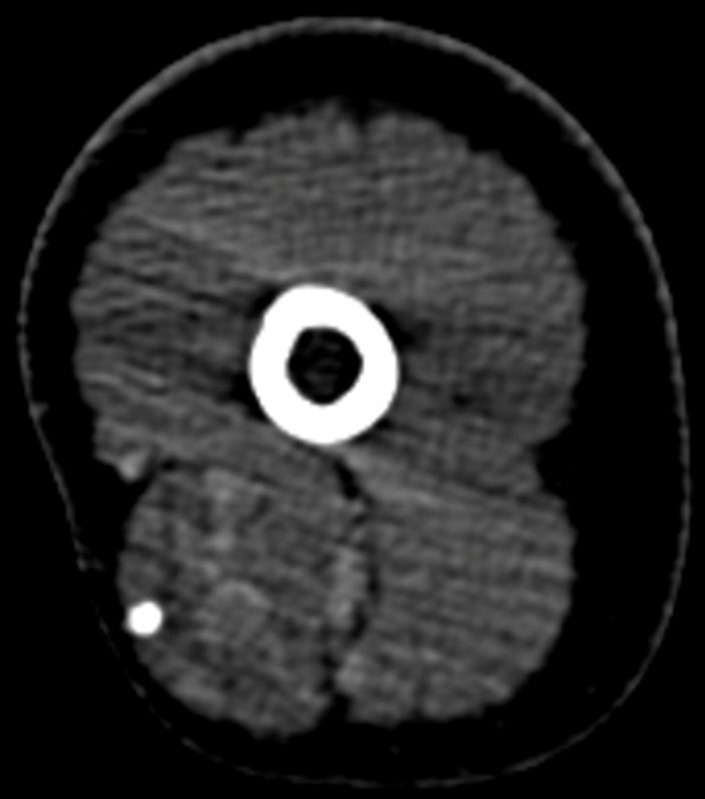
Post contrast CT 1 mm slice reconstruction in axial plane revealed the intramuscular tumors with calcification inside also with a lack of involvement of bone structures

We also performed a CT with contrast and an angioCT, which revealed the intramuscular tumors with calcification inside and also the lack of involvement of bone structures and of axillary structures (**Fig. 6**).

**Fig. 7:**
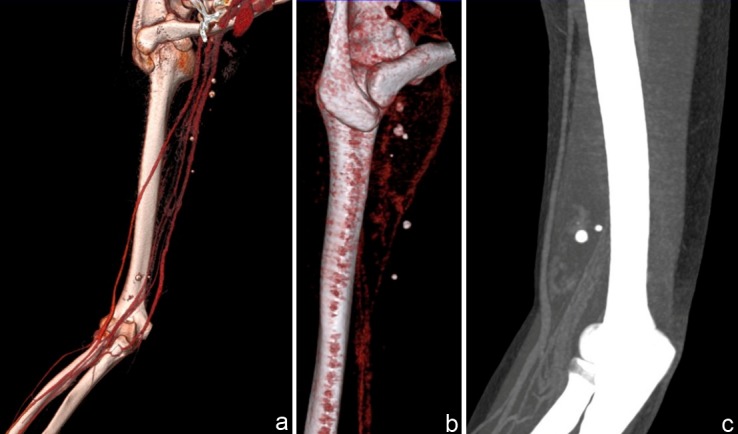
VRT (a,b) and MIP (7c) angioCT reconstructions indicating the integrity of arterial vessels and venous anomaly

We carried out maximum intensity projections (MIP) and volume-rendered reconstructions (VR) which indicated the venous anomaly and the integrity of the arterial vessels (**Fig. 7a,b,c**).

## Discussion

 Hemangiomas are the most frequent benign vascular neoplasm, being four times more frequent in females. Usually they appear as a single mass, but in a small percentage, they can be multiple. Hemangiomas develop in the head and neck, followed by the trunk and extremities [**4**-**6**]. The most common localization of intramuscular hemangiomas is the lower extremities, followed by the upper extremities. These soft tumors are often placed subcutaneously, and intramuscular extension is rare [**7**]. Cavernous hemangiomas are larger in size and deeper, with a high tendency in thrombosis and calcification [**8**].

 The case we presented enclosed the previously described characteristics, the lesion being discovered in a young female and localized in the upper limb, but unlike the other studies, the tumor extended to the brachial muscle fibers.

 Sinusoidal hemangioma is a rare variant of cavernous hemangiomas [**8**]. Pathologically, they are composed of dilated vascular type sinusoidal spaces, with focal atypia of the endothelium, which may lead to diagnostic confusion with vascular malformations, lymphangioma or a well-differentiated angiosarcoma [**9**]. In our patient, the use of immunohistochemical markers confirmed the vascular endothelial origin of the tumor and allowed a proper diagnostic thus making a differentiation from the other cases presented in literature.

Imaging diagnosis of these lesions is usually made by MRI and angiography. X-ray can reveal a nonspecific mass with calcification and bone changes if the tumor is located adjacent to the osseous structures [**10**]. The CT may identify a soft tissue mass with densities similar to muscles and also the phleboliths, even the small ones. The tumors enhance after contrast media administration, with depiction of serpentine vascular areas [**11**].

 The imaging evaluation of choice is MRI, this imaging technique being important in the assessment of these tumors. MRI is able to determine the extent in the surrounding tissue and accurately differentiate between vascular lesions with low-flow and high-flow [**12**,**13**].

The MR imaging usually exhibits a heterogeneous mass ofserpentine type, adjacent muscular atrophy and fat overgrowth. The MR features of these tumors are intermediate signal intensity on T1-weighted and high-signal intensity on T2-weighted images. A recent study reported that the cavernous type has a lobulated appearance, as in our case, in contrast with serpiginous aspect of capillary type [**14**]. The T1-weighted images may depict areas of high-signal-intensity adipose tissue due to chronic muscle atrophy as a result of chronic vascular insufficiency. The increased contrast media uptake demonstrates a high signal intensity tumor after gadolinium administration [**11**]. The need for multiple surgical interventions, as well as additional treatments can be managed by outlining if a lesion is focal, multifocal or diffuse by using MRI [**15**]. The presentation of our case was of focal lesion, which was treated by surgical removal in all recurrences as opposed to the last one with multifocal lesion in the arm. Also, the evolution of this type of hemangioma with numerous recurrences is rarely described in literature. The MRI topography of lesion is useful in assessing the involvement of neighboring structures, especially when percutaneous access is needed in order to reduce the risk of injury of nerves situated in the vicinity of vascular malformation. The depiction of the surrounding bones and muscles implication is helpful for the evaluation of the complication and post-procedure treatment [**16**]. The type of embolization may be guided by the characterization of lesions in low flow or high flow vascular disorders. The presence of signal voids is usually a feature of high-flow malformation and the absence of it characterizes the low flow lesions. In this case, the use of CT or plain X-ray determined the cause of signal void in the form of calcifications [**17**].

Recently angiography has probably been replaced by MR or CT angiography in providing anatomical vascular information, the main assignment for this imaging technique being the embolization prior to resection [**17**,**18**]. Vascular anomalies and mapping may be demonstrated by using CT angiography with multiplanar reconstructions (MPR), maximum intensity projections (MIP) and volume-rendered reconstructions (VR). CT angiography is a useful imaging technique in preoperative and postoperative evaluation of soft-tissue hamangiomas and also when MRI is contraindicated [**19**].

In this case, overlapped by numerous recurrence and multifocality, the therapeutical approach will probably take under consideration in the future other methods such as cryotherapy, electrocoagulation or laser therapy.

## Conclusions

The particularities of our case are represented by topography and histopathology of a rare type of cavernous malformation. The imaging techniques with the new post processing software along with the clinical data play an important role for diagnosis and evaluation of these lesions in order to avoid diagnostic pitfalls and also to correctly guide the treatment.
